# Dynamics of ColicinE2 production and release determine the competitive success of a toxin-producing bacterial population

**DOI:** 10.1038/s41598-020-61086-z

**Published:** 2020-03-04

**Authors:** Anna S. Weiß, Alexandra Götz, Madeleine Opitz

**Affiliations:** Center for NanoScience, Faculty of Physics, Ludwig-Maximilians-Universität München, Geschwister-Scholl-Platz 1, 80539 München, Germany

**Keywords:** Biophysics, Microbiology, Biological physics

## Abstract

The release of toxins is one mechanism used by bacterial species to establish dominance over competitors, but how the dynamics of toxin expression determine the competitive success of a toxin-producing population is largely unknown. Here, we investigate how the expression dynamics of ColicinE2 – a toxic bacteriocin – affect competition between toxin-producing and toxin-sensitive strains of *Escherichia coli*. We demonstrate that, in addition to genetic modifications in the toxin expression system, alterations of the growth medium can be used to modulate the timing of toxin production and the amount of toxin released. Thus cells that release the toxin at later times can accumulate more colicin. In experiments, we found that delaying toxin release does not significantly alter competition outcome. However, our theoretical analysis allowed us to assess the relative contributions of release time and toxin level to the competitive success of the producer strain, that might counteract each other in experiments. The results reveal that the importance of delaying toxin release lies in increasing the toxin amount. This is a more effective strategy for the toxin-producing strain than prompt discharge of the colicin. In summary, our study shows how the toxin release dynamics influence the competitive success of the toxin-producing bacterial population.

## Introduction

Ecological interactions between individual organisms govern ecosystem dynamics^1–4^ and determine the composition and stability of microbial populations^5–9^. Broadly speaking, ecological interactions may be either cooperative^10–13^ or competitive^4,9,14,15^ and are mediated by various mechanisms^3,16,17^. One well-known type of competitive bacterial interaction involves the lysis-dependent release of toxin(s) directed against closely related species^18–20^, which can be accompanied by a division of labour between toxin-producing and reproducing cells within a clonal population^21^. Despite the growing knowledge of toxin competition mechanisms, it remains unclear, how the dynamics of toxin expression, most importantly the timing of toxin release, influence competition between toxin producers and toxin-sensitive bacteria.

**Figure 1:**
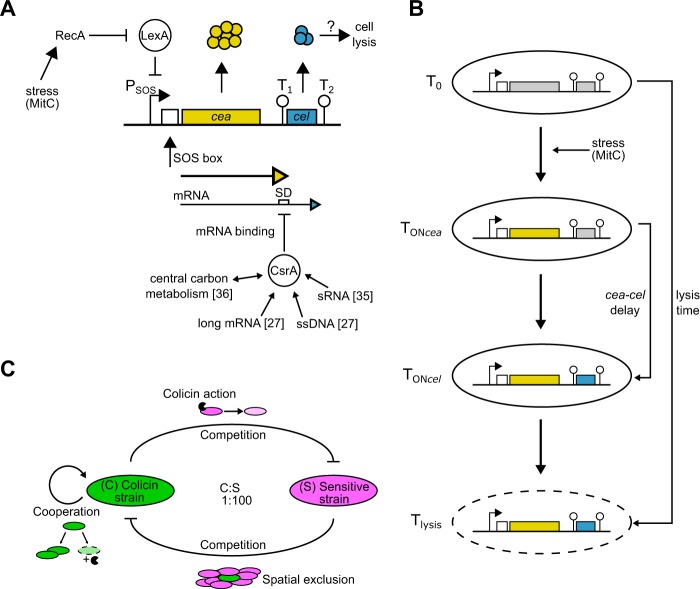
ColicinE2 expression dynamics and competition between a toxin-producing and a toxin-sensitive strain. (**A**) Schematic depiction of the regulatory network that controls the synthesis of ColicinE2. (**B**) Sequence of gene expression steps and cell lysis. Time-point T_0_: no expression, T_ON*cea*_: onset of *cea* expression, T_ON*cel*_: onset of *cel* expression, T_lysis_: continuing synthesis of both gene products and cell lysis. (**C**) Interaction between a toxin-producing strain C and the sensitive strain S is characterized by interstrain competition (spatial exclusion and toxin action) and intrastrain cooperation between members of the C strain population^21^.

The bacteriocin expression system used in our experimental setting is the well-studied ColicinE2 system in *Escherichia coli*^22–25^. This bacterial toxin expression system provides a paradigmatic model to dissect the dynamics of toxin production and toxin release by cell lysis in a situation in which a ColicinE2-producing strain C competes with a toxin-sensitive strain S (Methods, Supplementary Table 1, Fig. 1). In this context, the transcriptional and post-transcriptional circuits controlling ColicinE2 expression dynamics are well understood^19,26,27^.

ColicinE2 is heterogeneously expressed from the ColicinE2 operon on the pColE2-P9 plasmid^26,28,29^ upon induction of the noisy bacterial SOS response^28,30,31^. Expression can therefore be triggered by exposure to the antibiotic mitomycin C (MitC)^32^, with higher MitC levels increasing the fraction of toxin producers within the C population^26^ (Fig. 1a). Induction of the SOS response leads to the production of two mRNAs from the ColicinE2 operon: a short transcript that includes the *cea* gene (encoding the colicin) and the *cei* gene (the immunity gene), and a long transcript that comprises the entire ColicinE2 operon, and therefore includes the *cel* gene (the lysis gene) in addition to *cea* and *cei* (Fig. 1a)^19^. The RNA-binding protein CsrA^33,34^ inhibits translation of the *cel* gene, thus preventing immediate toxin release by cell lysis. Hence, as long as sufficiently high levels of free CsrA molecules are present, synthesis of the lysis protein is inhibited and no toxin can be released. The abundance of CsrA is in turn regulated by CsrA-sequestering elements, such as the sRNAs CsrB and CsrC^35^, the long mRNA produced from the ColicinE2 operon^27^ or the ssDNA originating from autonomous rolling-circle replication of the pColE2-P9 plasmid^27^. In addition, the amount of CsrA within a given bacterial cell is coupled to the metabolic state of that cell^36^.

Although the network that regulates expression of ColicinE2 is well understood, and quantitative data on its expression dynamics as a function of the external stress level are emerging^26,27^, the role of the timing of toxin production and lysis protein synthesis in the competitive success of the toxin-producing population remains poorly understood (Fig. 1B,C). In particular, it is not clear whether a late toxin release correlating with a long delay between the onset of *cea* and *cel* expression (=*cea-cel* delay in Fig. 1B) is beneficial for the toxin producer. In addition, the impact of metabolites on ColicinE2 expression dynamics (especially toxin release times) and the competitive success of the toxin-producing population remains largely unexplored.

In this study, we first investigated ColicinE2 expression dynamics, and in a second step how the dynamics influence competition between a toxin producer C and a toxin-sensitive strain S (Fig. 1c), when both are grown on either glycerol or glucose as carbon source. This approach enabled us to analyse the importance of the delay between ColicinE2 production and release for the competitive success of the toxin producer. We found that toxin-producing cells grown on glucose undergo cell lysis at later times than cells grown on glycerol. In addition, strains that release the toxin at late time-points also released larger amounts of toxin. Both factors, together with the respective growth rates of the particular strains present in a competitive setting are found to determine the outcome of the competition.

## Results

### Post-transcriptional regulation via CsrA affects toxin expression dynamics

To elucidate how the dynamics of toxin expression influence bacterial competition, we first performed a detailed analysis on these dynamics using fluorescence time-lapse microscopy (Methods). In order to monitor *cea* and *cel* gene expression, we added a reporter plasmid on which the toxin and lysis genes were replaced by sequences encoding the Yellow and Cerulean Fluorescent Proteins (YFP and CFP), respectively (Methods). This allowed us to manipulate the timing of toxin production and release systematically. In the current study, we vary the interval between the onset of *cea/yfp* expression (toxin production) and the induction of the *cel/cfp* expression (whose product mediates cell lysis and toxin release) by shifting the initiation of *cel/cfp* expression (T_ON*cel*_). In a previous study^27^, we demonstrated that the duration of this *cea-cel* delay (Fig. 1b) depends on the availability of the RNA-binding protein CsrA, which post-transcriptionally inhibits Cel synthesis. Here, multiple CsrA-sequestering elements (sRNA, long mRNA, ssDNA) control the abundance of free CsrA (Fig. 1a). The amount of long mRNA produced is in turn dependent on the copy number of the toxin-producing pColE2-P9 colicin plasmid and/or reporter plasmids carrying a CsrA-binding site^27^. Consequently, reducing the plasmid copy number of the reporter plasmid by changing the origin of replication^27^, enabled us to create two toxin-producing strains that differ in the duration of their *cea-cel* delay (C_REP1_ and C_REP2_ carrying pMO3 and pMO8, respectively; see Supplementary Table 1). C_REP1_ expresses *cea* and *cel* nearly simultaneously, while C_REP2_ has a significantly prolonged *cea-cel* delay^27^ in comparison to C_REP1_. In the wild-type strain C_WT_, the *cea-cel* delay cannot be determined experimentally, as any genetic changes on the native pColE2-P9 plasmid alter the natural expression dynamics. However, in a previous study^27^, we were able to theoretically estimate the length of the delay in C_WT_ as being on the order of 1 h. Moreover, it is not possible to significantly extend the *cea-cel* delay in C strains beyond the 60 min determined for C_WT_ without changing the native colicin plasmid, as the CsrA binding site of the *cel* gene is nearly optimal^37^ and therefore stronger binding of CsrA is hardly achieved. For a detailed analysis of how CsrA availability controls the duration of the *cea*-*cel* delay, we refer the reader to Goetz *et al*.^27^. Consequently, in this study we focus on the strains C_REP1_ and C_REP2_ that have a shorter *cea-cel* delay than C_WT_.

**Figure 2:**
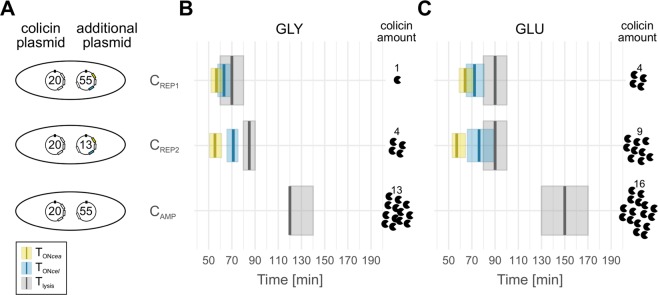
Post-transcriptional regulation via CsrA affects toxin expression dynamics. (**A**) Schematic depiction of the different toxin-producing strains with their respective plasmid composition (Supplementary Table 1). Numbers inside the plasmids indicate the plasmid copy number. (**B,C**) Times of onset of *cea* expression (T_ON*cea*_ = yellow), *cel* expression (T_ON*cel*_* = *cyan) and cell lysis (T_lysis_ = grey) for toxin-producing strains grown on glycerol (**B**) or glucose (**C**). Thick lines show the respective median, the shaded area indicates the interquartile range. Furthermore, the relative amounts of toxin released by each strain compared to the amount released by C_REP1_ on glycerol are given. These toxin amounts are determined from the experiment described in Supplementary Fig. 1).

In a first experiment, we investigated the ColicinE2 expression dynamics for C_REP1_ and C_REP2_ grown on the exact same growth medium as required for competition experiments with glycerol as a standard carbon source (Methods) and investigated *cea/yfp* and *cel/cfp* expression as well as the time-point of cell lysis. As shown in Goetz *et al*.^27^, the duration of the *cea-cel* delay upon SOS induction is independent of the MitC concentration. In the present work, a high MitC concentration of 0.25 µg/µl was used to induce the SOS response, which ensures that nearly all cells switch into the toxin-producing state and sufficient numbers of cells are available for data analysis. We found that C_REP1_ has a short *cea-cel* delay of about 6 min at these growth conditions, with cell lysis occurring at 70 min after addition of MitC (Fig. 2a,b; results of the single-cell analysis can be found in the SI Data Table). The interquartile range for all expression dynamics data is shown in Fig. 2. The results of the significance analysis are given in the Supplementary Table 4. In C_REP2_ the mean length of the *cea*-*cel* delay is 19 min, and cell lysis ensues at 85 min after induction with MitC (Fig. 2a,b). Therefore, C_REP1_ and C_REP2_ show clear differences in their toxin expression dynamics.

CsrA abundance has also been shown to be influenced by the cell’s metabolic state^36^, since CsrA is strongly interconnected with central carbon metabolism (Fig. 1a), acting as a positive regulator of glycolysis and suppressor of glycogenesis. Consequently, carbon sources such as glycerol and glucose affect CsrA levels differently, by changing fluxes through central metabolic pathways. Hence, we hypothesized that changing the available carbon source should also have an impact on the kinetics of toxin expression. We therefore repeated the fluorescence time-lapse experiments on both strains during growth on glucose as carbon source. In this case, we found a slight increase in the duration of the *cea-cel* delay in both strains, and lysis times are shifted to later time-points (Fig. 2c). Specifically, while the interval between the activation of *cea* (T_ON*cea*_) and cell lysis (T_lysis_) is 15 min and 25 min for C_REP1_ and C_REP2_ grown on glycerol, the corresponding values for C_REP1_ and C_REP2_ grown on glucose are 25 min and 35 min, respectively. Taken together this demonstrates that the nature of the carbon source indeed has an effect on toxin expression dynamics.

To further obtain insights into the behaviour of the natural expression dynamics of ColicinE2, we investigated a third C strain, C_AMP_ (Methods, Supplementary Table 1). We chose this strain instead of the wild-type C_WT_ for the long-term competition experiments described in the next section, because – unlike C_WT_ – it contains an antibiotic resistance marker that can be used to prevent contamination. However, as C_WT_, strain C_AMP_ does not carry a fluorescent reporter plasmid, so that *cea* and *cel* expression cannot be monitored. We found that C_AMP_ undergoes lysis 120 min after induction with MitC on glycerol and 150 min on glucose (Fig. 2). These late lysis times are in a similar range as obtained for C_WT_ in a previous study^27^.

Overall, we found that toxin-producing strains grown on glucose undergo lysis significantly later than when glycerol is used as the carbon source. In addition, compared to C_REP1_ toxin-producing cells grown on glycerol, cells in which lysis is significantly delayed can accumulate and release larger amounts of toxin into the surrounding medium (Fig. 2). Specifically, we found a linear correlation between the lysis time and the levels of toxin released into the environment by a particular C strain (R^2^ = 0.89, Supplementary Fig. 1).

### Two-strain competition on different carbon sources

Having characterized the duration of the *cea*-*cel* delay and the times of lysis in the two C_REP_ strains grown on different carbon sources, we went on to examine how the differences in toxin-expression dynamics influence the competitive success of the toxin-producing population. To address this question, we investigated two-strain competitions between one or other of the toxin-producing strains described above (and referred to as C_X_ in the following, Supplementary Table 1) and a single toxin-sensitive strain S. These strains were plated at an initial C_X_:S ratio of 1:100 (see Supplementary Table 1) and grown for 48 h on solid media containing either glycerol or glucose as carbon source (Fig. 1c, Methods). The C_X_:S competition is characterized by indirect intrastrain cooperation between reproducers and toxin producers within the C strain population and interstrain competition between the C and S strains, mediated via toxin action and denial of access to resources by spatial exclusion. The latter is facilitated by the initial strain ratio, which favours the S strain, thus boosting its competitiveness. Clearly, varying the initial strain ratio will have an effect on the outcome of the competition^25^. However, the initial C_X_:S ratio of 1:100 chosen here is based on earlier studies demonstrating that coexistence of the two strains is only possible at small C strain fractions^21,25^.

**Figure 3:**
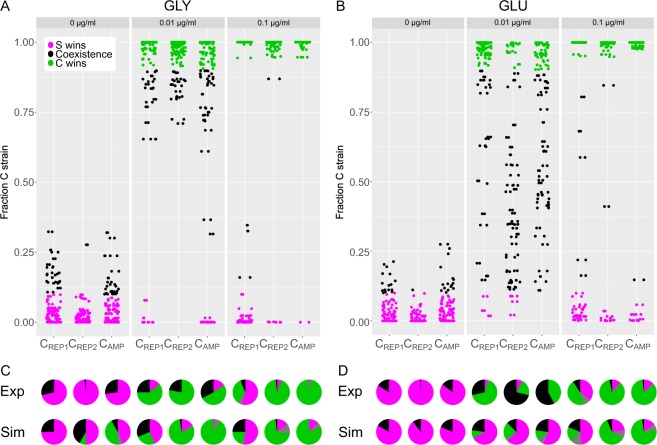
Competition between toxin-producing strain C and toxin-sensitive strain S on different carbon sources. Final fraction of C_X_ after competition (dot plot, **A,B**) and classified outcomes (pie plots, **C,D**) for competition on glycerol (**A,C**) or glucose as carbon source (**B,D**). Results are presented as a function of the external inducer concentration MitC (indicated at the top of the dot plots) for the three different C_X_:S competitions. In (**C,D**) also the outcome of the competition in the numerical simulation (bottom row) is given. (**C,D**) Outcome fractions in pie charts are given as S wins (magenta, <10% C), coexistence (black, 10–90% C), C wins (green, >90% C) and extinction (grey, no bacteria detected (C or S). Extinction is due to the killing of S cells by the action of C’s toxin and loss of C cells due to lysis.

We studied the C_X_:S interaction for three different levels of external stress, thereby tuning the fraction of C cells that produce the toxin^26^ (Fig. 3). We found that in the absence of external stress (0 µg/µl MitC), when only small fractions of the C_X_ strain populations actually produce the toxin^26^ (Supplementary Fig. 2), the sensitive strain is usually able to outcompete the toxin-producing strain as a result of spatial exclusion. However, in the presence of external stress (0.01 and 0.1 µg/µl MitC), when higher fractions of the C_X_ strain populations produce and release the toxin, the C strain was able to dominate the competition (Fig. 3) in most cases. Upon comparing C_X_:S competitions on glycerol versus glucose, we detected only one major difference – namely that, when grown on glucose, S and C strains were able to coexist in many competitions at the intermediate MitC concentration of 0.01 µg/µl (Fig. 3).

Most importantly, we found that competition outcome was independent of the C_X_ strain studied, although the strains differ in their respective *cea-cel* delay. This result indicates that varying the time-point of toxin release does not have a significant effect on the competitive success of the C strain or that unknown compensatory effects come into play.

### Theoretical Modelling disentangles the factors determining competition outcome

Varying the time-point of toxin release has two important consequences: (i) if the toxin is released at later time-points, the competing S strain population has more time to expand, and (ii) late release allows the C strain to accumulate the toxin over a longer period, such that cell lysis results in the release of a larger amount of toxin into the medium.

Even though both of these factors differ for the individual C strains (Fig. 2), the data shown in Fig. 3 demonstrate that neither of them has any measurable effect on the outcome of our competition assay. Therefore, in order to disentangle the impact of the two above described factors, which cannot be distinguished in competition experiments, we set up a theoretical model of the competition scenarios (Methods, Supplementary Fig. 3a). We used a stochastic lattice-based model, based on the model described in Bronk *et al*.^21^, which allows us to explicitly incorporate the stochastic positioning and phenotypic heterogeneity of the C strain. A schematic of the strain interactions captured by the model is given in Supplementary Fig. 3a. In the model, phenotypic heterogeneity is a consequence of stochastic switching^31^ from the reproducing state ***C*** to the toxin-producing state ***C***_***on***_. Due to the fact that toxin release is coupled to cell lysis, producing cells can only decay and cannot switch back to the reproducing state (Methods, Supplementary Fig. 3). We performed numerical simulations of the C_X_:S competitions with parameters obtained from our experiments (Supplementary Figs. 2 and 3 and Supplementary Table 3). We found that the simulation outcomes generally retrieved the main type of competition outcome observed in our experiments (S wins, C wins, coexistence or extinction) for high external stress levels and in the absence of external stress (Fig. 3c,d, for quantitative data see Supplementary Table 6). However, for intermediate external stress levels we observed a strong discrepancy between the experimental findings and simulations. Experiments as performed in this study are inherently noisy and particularly sensitive to the initial conditions. Furthermore, at intermediate stress levels stochastic variation in toxin expression and release plays an important role that cannot easily be incorporated into simulations. Hence, we believe that the observed differences between the experimental data and the numerical simulations at intermediate MitC concentrations largely originate from the heterogeneity in toxin expression that is strongest at intermediate stress levels^26^.

**Figure 4:**
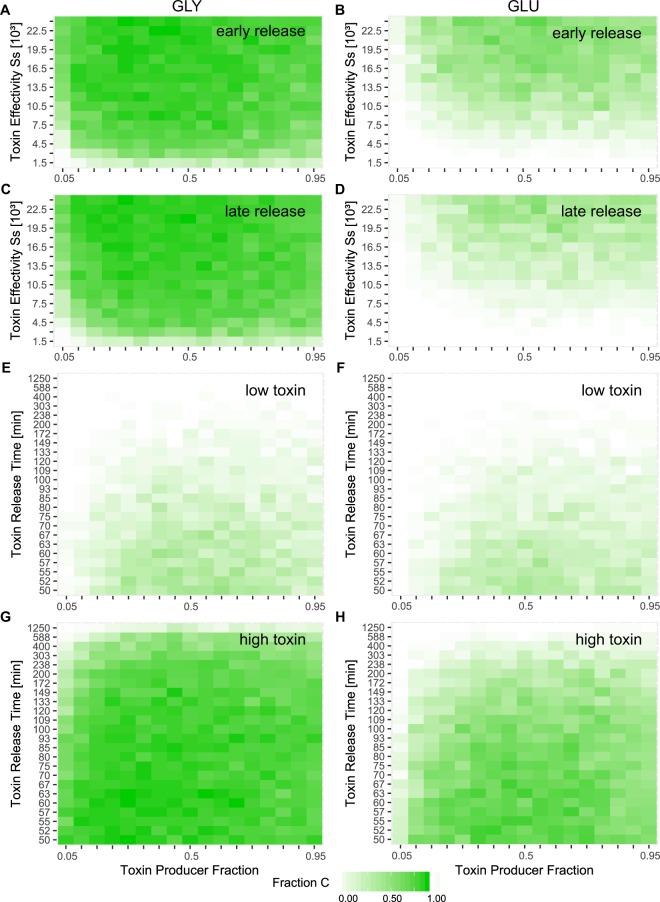
Numerical simulations demonstrate the importance of the amount of toxin released (**A–D**) and the effects of delaying its release (**E–H**) for the competitive success of the C strain. For different conditions, parameter sweeps were performed and the fraction of the C_REP1_ strain that survives at 48 h is plotted. Left column: growth rate of C_REP1_ on glycerol, right column: growth rate of C_REP1_ on glucose-supplemented medium. Toxin release time: 68 min (**A**), 90 min (**B**), 120 min (**C**) and 149 min (**D**). (**A–D**) Parameter sweeps for different toxin effectivities *s*_*S*_. (**E**) *s*_*S*_ = 1500, (**F**) *s*_*S*_ = 6000, (**G**) *s*_*S*_ = 19500, (**H**) *s*_*S*_ = 24000. (**E–H**) Parameter sweeps for varying toxin release times. The data showing the fraction of S in the competition and Coexistence are given in Supplementary Figs. 4 and 5.

However, taken together, the results of our theoretical model are generally compatible with the overall outcome of our competition experiments (Supplementary Table 6). Consequently, we used this model to further disentangle the role of the different factors correlated with toxin release. We performed parameter sweeps to investigate the impact of the amount of toxin released as well as the importance of the time-point of cell lysis on two-strain competition (Fig. 4). In the theoretical model, the amount of toxin released is incorporated into the parameter ‘toxin effectivity’, *s*_S_ = *σ*_*S*_ · *n*_*tox*_, which is composed of two terms – the toxin sensitivity of the S strain (*σ*_*S*_), which remains constant in this study and the amount of toxin released (*n*_*tox*_) representing the amount of toxin produced by cells in the *C*_*on*_ state. Hence, the precise magnitude of the parameter ‘toxin effectivity’ is difficult to determine experimentally. In this study we use *s*_*S*_ = 1500 unless stated otherwise (with *σ*_*S*_ = 1500*, n*_*tox*_ = 1). This value is based on the previous analysis of the C:S interaction described in Bronk *et al*., 2017^21^. Furthermore, the relative amounts of toxin released by the different C_X_ strains (Fig. 2 and Supplementary Fig. 1) were directly incorporated into the model. We found that irrespective of the fraction of C cells that produce the toxin, the C strain wins the competition as long as large amounts of toxin can be synthesized and released (*s*_S_ > 1500, *n*_*tox*_ > 1 in our simulations, Fig. 4a–d). This finding holds for simulations of C_X_:S competitions on both glucose and glycerol, and is not dependent on the time-point of cell lysis (Fig. 4a–d). However, in competitions on glucose, a higher amount of toxin must be released by the C strain in order to be effective, as the S strain itself exhibits a significantly higher growth rate than the C_X_ strains, unlike the case on glycerol (Supplementary Fig. 2, SI Data). The second important factor, the time-point of lysis in the C_X_ strains, is given in the model by *1/d*_*Con*,_ with *d*_*Con*_ being the lysis rate of toxin producers. Here, when amounts of toxin released by cell lysis are low, the C strain can only outcompete the S strain if the toxin is released at early time-points by intermediate fractions of toxin-producing cells within the C strain population. If large amounts of toxin are produced, C wins the competition in most of the cases (Fig. 4e–h). Taken together, our theoretical analysis clearly showed that, although the amount of toxin released is directly correlated with the time-point of cell lysis (experimental data: Supplementary Fig. 1b, R^2^ = 0.89), with late cell lysis releasing more toxin, the effect on two-strain competition is not linear, and the two factors differ in their respective impacts on competition outcome. Of the two, increasing the amount of toxin released turns out to have the greater effect on the C:S competition.

## Discussion

In this study, we investigated in a combined experimental and theoretical analysis how ColicinE2 expression dynamics affect competition between a toxin-producing population and toxin-sensitive bacteria. We first investigated the kinetics of toxin expression in three closely related colicin-producing strains (C_REP1_, C_REP2_ and C_AMP_), which differ in their genetic background (Material and Methods, Supplementary Table 1) in ways that affect the timing of toxin production and toxin release (Fig. 2). In particular, these strains show differences in the duration of the delay between the onset of expression of the genes *cea* (which encodes the toxin) and *cel* (whose product triggers toxin release). Furthermore, we demonstrated that toxin expression dynamics are also influenced by the carbon source (glucose or glycerol) used, and that if cell lysis occurs at later time-points, larger amounts of the toxin are released into the environment. The exact mechanism of ColE2 release by cell lysis has not been fully elucidated^19,38^. However, Pugsley *et al*.^22,39^, provided evidence that the lysis protein (a small lipoprotein) encoded by the *cel* gene of the ColE2 operon induces the permeability of the cell envelope, thus enabling toxin release. Since cell lysis takes place at later time-points when toxin-producing strains are grown on glucose, we hypothesize that bacterial cells grown on this energy-rich carbon source have a greater chance of repairing the cell envelope, thus delaying cell lysis. However, more experimental evidence is needed to elucidate the exact mechanism of ColicinE2 release.

We then investigated the impact of the differences in toxin expression dynamics between the three colicin producers on the outcome of competition with the toxin-sensitive S strain. Interestingly, although the three toxin producers differ in the duration of their *cea-cel* delay and consequently their time-point of toxin release by cell lysis, we found no significant differences in outcomes of competitions between the three strains initiated under various levels of external stress and on either carbon source.

These experimental results were confirmed by our theoretical modelling for high stress levels and in the absence of stress. At intermediate stress levels, we found a strong discrepancy between the experimental data and the numerical simulations. This is largely attributable to the inherently noisy nature of the experiments, which cannot be incorporated into the theoretical analysis. An additional factor contributing to the observed differences between experiment and simulation, which is not accounted for in the theoretical model, is the ability of the ColicinE2 to induce its own production^40,41^. However, the theoretical analysis retrieved the same general trends as our experimental observations, and further allowed us to investigate the importance of later cell lysis for the competitive success of the toxin producer. Specifically, it enabled us to disentangle the influence of two factors that are correlated with cell lysis and coupled in nature: the amount of toxin produced and the toxin release time. We believe that these two factors counteract each other. On the one hand, later C cell lysis allows the S strain to expand for a longer time. On the other hand, late cell lysis enables the C strain to release higher amounts of toxin, however, at late time-points. Consequently, these different factors could effectively compensate for one another, ultimately leading to the same competition outcome.

On varying toxin release times and toxin amounts over broad ranges in numerical simulations we found that indeed the C strain was only able to win in our simulations for low toxin amounts if the toxin was released early on. However, if high toxin amounts were released, the C strain won the competition in most cases, even if cell lysis occurred at late time-points. Furthermore, our theoretical investigation showed that doubling the amount of toxin released (e.g. on glycerol) has a very strong effect, while further increases have little effect on competition outcomes (Fig. 4). Consequently, we conclude that, at early time-points, a delay in toxin release ensures that a significant amount of toxin is produced. Once this is assured, delaying toxin release further has no marked effect, although it does prevent premature cell death and toxin release to no purpose. This might be the case under optimal nutrient conditions (such as growth on glucose), even if an antibiotic stress is present. Both strategies may be important for the wild-type toxin-producing strain C_WT_. Like the C_AMP_ strain used in this study, which is genetically most close related to C_WT,_ the wild-type strain produces a large amount of colicin (Supplementary Fig. 1) and releases the toxin at very late time-points (~150 min)^27^.

In summary, our results show how differences in toxin expression dynamics affect the competition between a toxin-producing population and toxin-sensitive bacteria. Furthermore, our findings elucidate how a delay in toxin release benefits the toxin-producing population, if the toxin is released by cell lysis, as is the case for groupA colicins^19^. GroupB colicins however, do not possess a lysis protein gene^19^. Here, toxin release is achieved by cell lysis that is induced through the presence of temperate phages^42,43^. Consequently, toxin expression dynamics of groupB colicins differ from those described for ColicinE2 in this study, as do the expression dynamics of other colicins, such as colicin Js or the recently described colicin Z^44,45^. In the case of colicin Js, the lysis gene is located upstream of the colicin structural gene^45^. Nevertheless, our study emphasizes the importance of toxin expression dynamics and that the precise timing of toxin release might be a relevant biological trait in the context of bacterial competition.

## Materials and Methods

### Bacterial strains and culture

The bacterial strains used in this study are listed in Supplementary Table 1. The toxin-sensitive S strain (S_RFP_) carries the plasmid pBAD24-mCherry for permanent induction of red fluorescence by the sugar arabinose, which enables the S strain to be distinguished from C_X_ strains in competition experiments.

The strain C_WT_ represents the original wild-type strain, which carries only the toxin-producing plasmid pColE2-P9. The C_REP1_ strain was constructed as described by Mader *et al*.^26^ and carries the double reporter plasmid pMO3. This plasmid enabled us to clearly distinguish toxin-expressing cells from cells that produced and released the toxin at basal levels or not at all. Furthermore, pMO3 harbours the entire ColE2 operon, in which the genes *cea* and *cel* have been replaced by genes coding for the fluorescent proteins mVenus (YFP) and mCerulean (CFP), respectively (Fig. 1a). This plasmid retains all regulatory sequences relevant for the binding of LexA to the SOS box of the ColE2 operon, and of CsrA to the Shine-Dalgarno sequence (SD) on the resulting long mRNA. In addition, C_REP1_ carries the wild-type toxin-producing plasmid pColE2-P9 found in C_WT_. C_REP2_ was created as described in Goetz *et al*.^27^. This strain is identical to C_REP1_, except that the copy number of pMO3 is reduced from 55 to 13 by changing the ORI of pMO3, resulting in plasmid pMO8.

The wild-type strain C_WT_ does not carry an antibiotic resistance, which however is necessary for the long-term competition experiments performed in this study. We therefore constructed a third strain, called C_AMP _, that carries pColE2-P9 (ensuring that wild-type regulation of ColE2 expression is retained) and an additional plasmid bearing an ampicillin resistance gene required for long-time competition experiments. The strain C_AMP_ was created as follows: Using the primers P1 and P2 (Supplementary Table 2), a PCR with the pMO3 plasmid was performed to eliminate the ColE2 operon, while retaining the backbone of the pMO3 plasmid with the ampicillin resistance. The resulting DNA fragment was cut with the enzymes KpnI and DpnI (NEB), then ligated and transformed into strain XL1 by electroporation. The cultures were grown in SOC medium for 1 h and then grown overnight on selection plates with LB and ampicillin. After sequencing, the plasmid was further transformed into the C_WT_ strain, creating the new strain C_AMP_.

### Liquid cultivation and media

Bacterial cultures were grown in M63 minimal medium supplemented with either glycerol or glucose as carbon source. The nature of the available carbon source was expected to have an impact on the Csr system in the bacterial cell, especially on the abundance of free CsrA molecules, thus affecting the post-transcriptional repression of the lysis gene (*cel*) in the ColE2 operon. The amount of glycerol or glucose added to the medium was adjusted to ensure that both media contained the same amount of carbon.

For the experiments, liquid overnight cultures were diluted to OD_600_ = 0.1 in medium supplemented with 100 µg/ml ampicillin, 0.2% arabinose and either glycerol or glucose as carbon source. Bacteria were then grown to OD_600_ = 0.2 and again diluted to OD_600_ = 0.1 for further use in competition and other experiments.

### Fluorescence time-lapse microscopy and data analysis

Single-cell fluorescence time-lapse microscopy and general data analysis were performed as described earlier^27^. These analyses were conducted at an external stress level of 0.25 µg/µl MitC, as this level of inducer ensures that nearly all C cells switch to the toxin-producing state *C*_*on*_^26^. The time-point T_ON_ marks the onset of the ‘ON’ state, and is defined as the time at which single-cell fluorescence exceeds a switching threshold^27^ for *cea* and *cel* gene expression (T_ON*cea*_ and T_ON*cel*_, respectively) following induction with MitC. The duration of the delay between onset of *cea* and *cel* expression was calculated as the mean of the T_ON*cel*_ - T_ON*cea*_ values for individual cells expressing both *cea/yfp* and *cel/cfp*. The time-point of cell lysis corresponds to the time elapsed after the addition of MitC. Statistical data analysis was performed and plots were generated using the programming language R (Version 3.5.2) and R Studio (Version 1.1.463). All figures presented in this manuscript were created using Inkscape (Version 0.91).

### Competition experiments and data analysis

Range expansion competition experiments were performed over a period of 48 h using a multi-scale set-up described earlier^21^, which allowed us to monitor up to 77 competition experiments in parallel. Aliquots (5 nl) of the inoculum culture were deposited on the experimental plate by a Labcyte Echo 550 Liquid Handler using acoustic droplet ejection as described in Bronk *et al*.^21^,. Experiments were repeated 2–3 times at an C_X_:S ratio of 1:100. Only communities containing C cells in the initial colonies were analysed, resulting in a minimum of 95 competitions per experimental condition. To obtain the individual strain growth rates for each competition, single strain spots were inoculated in parallel to the two-strain competitions on the same plate. Image and data processing was performed as described in detail in Bronk *et al*.^21^, using Mathworks MATLAB software (Version 2017b) and the statistical programming language R (Version 3.5.2) and R Studio (Version 1.1.463), and plots were combined with Inkscape (Version 0.91). Growth rates of the single-strain colonies were obtained in the linear area growth regime by linear fitting (see below). In competition experiments, we observed four distinct outcomes based on the relative area occupied by a particular strain: domination by C or S, coexistence, and extinction of both strains. Domination is defined as the occupation of over 90% of the colony area by one strain, coexistence denotes occupancies of between 10% and 90%, and occupation of the total area of less than 2.4*10^4^ µm^2^ constitutes extinction.

### Determination of growth rates

Growth rates of the S and C_X_ reference strains (single strain spots as described above) were analysed manually using ImageJ and a graphic tablet (Wacom Intuos Art M) by marking the area occupied by each colony from 11 to 48 h of incubation. The resulting growth curves were then plotted with Igor Pro (Version 7.04) and subjected to linear fitting for the period 20–48 h.

### Determination of toxin amounts produced by a particular C strain

To test the influence of Colicin E2 on the growth behavior of the S strain, colicin was extracted from a MitC-induced C_X_ culture. Therefore, C_X_ cultures were grown as described above. The dilution to OD_600_ = 0.1 was then supplemented with 0.25 µg/ml MitC and incubated for 160 min to ensure that most cells switch to the colicin-producing state and subsequently release the colicin by cell lysis into the medium. During the incubation of the C_X_ strains, a culture of the S strain was grown to OD_600_ = 0.2 and then diluted to OD_600_ = 0.1, and 500-µl aliquots were streaked out as a thin, but evenly distributed film onto warm M63 agar plates, supplemented with 100 µg/ml ampicillin and 0.2% arabinose. These plates were incubated for at least 1.5 h at 37 °C to ensure even and dense S cell growth before the colicin was applied onto this S cell base. After incubation, the induced C_X_ culture was centrifuged for 15 min at 13 krpm to remove cell debris. To extract the colicin, 500 µl of the supernatant was filtered through 10-kDa filters (Amicon Ultra 0.5 ml). The concentrated colicin solution was then diluted 1000fold, and 50-nl aliquots were deposited on the experimental plate using the same method as for competition experiments, resulting in an average spot area of 2 mm². On every experimental plate, colicin extracts obtained from all four C strains (C_REP1_, C_REP2_, C_AMP,_ C_WT_) were tested.

The experimental plates were analysed using a fluorescence microscope (SMZ25, Nikon) set-up by taking a brightfield and RFP image immediately after transferring the colicin dilution and again after 16 h of incubation at 37 °C. Depending on the colicin concentration in the sample, S growth is distinctly altered in the spot area, and these areas were analysed using ImageJ for each individual spot.

### Live-dead screening of bacterial strains

For the live-dead screening, bacteria were grown as described above. Day cultures were grown to an OD_600_ = 0.2 and passed through 100 K filters (Amicon Ultra 0.5 ml) to remove already lysed cells and toxin. Cultures were then adjusted to OD_600_ = 0.1 and induced with MitC concentrations of 0.00 µg/ml; 0.01 µg/ml and 0.10 µg/ml for 3 h. After induction, 50 µl samples of cells were stained with 0.5 µl of mixed dye (1:1 ratio of SYTO 9: propidium iodide) for 15 min (LIVE/DEAD BacLight Bacterial Viability Kit, ThermoFisher Scientific). The stained cells were then transferred onto an agar plate and analyzed with an upright fluorescence microscope (90i, Nikon). Image analysis was performed manually by counting red and green cells using ImageJ.

### Modelling and simulations

A stochastic lattice-base computational model was used to simulate competition between the C_X_ strains (C_REP1_, C_REP2_ and C_AMP_) and the toxin-sensitive strain S as described in Bronk *et al*.^21^,. Initial communities used for simulations were created in accordance with experimental conditions, starting with random spatial distributions of C_X_ and S cells in an approximate 1:100 (C:S) ratio within a circular field, each containing at least one initial C_X_ cell. Initial colony density was chosen in accordance with experimental conditions. Five different species of agents were used in this model: viable C_X_ and S cells, colicin-producing *C*_*on*_ cells, growth-inhibited S_stop_ cells, and unoccupied agar sites A (Supplementary Fig. 3). Reactions were modelled using a Moore neighborhood (8 nearest neighbors), where the rates for diagonal growth were scaled by a factor $1/\sqrt{2}$. Possible reactions comprised reproduction of viable C_X_ and S cells, C_X_ cells switching to a producing *C*_*on*_ state with the switching rate s_c_, subsequent lysis of the *C*_*on*_ cell with concomitant colicin release, and transition of S cells to a growth-inhibited *S*_*stop*_ state cells in response to the action of colicin (Supplementary Fig. 3). As soon as colicin was released by a lysing *C*_*on*_ cell, an exponential colicin profile was assumed to originate from this position, as described previously^25^. Model parameters and reaction rates are given in Supplementary Table 3 and the SI Data. The lysis rate of *C*_*on*_ cells (*d*_*Con*_) was adjusted according to the experimental data obtained for each particular C_X_ strain. The remaining free parameter, toxin effectivity ${s}_{S}={\sigma }_{S}\cdot {n}_{tox}$ is composed of two terms, the toxin sensitivity of the S strain ${\sigma }_{S}$ and toxin amount factor ${n}_{tox}$ representing the amount of toxin produced by ${C}_{on}$. As described in Bronk *et al*.^21^, a toxin sensitivity of ${\sigma }_{S}=1500$ and toxin amount factor ${n}_{tox}=1.0$ were chosen as ‘standard conditions’ for C_REP1_:S competition on glycerol. As our experiments had shown (Supplementary Fig. 1) that the amount of toxin released varied between the different C_X_ strains grown on the two different carbon sources, the toxin amount factor was adjusted accordingly for each competition condition.

Simulations of the competition were performed in 48 rounds with 2970 time-points on a 250 × 250 lattice. Coarse graining was performed when an edge came into contact with a colony, and the simulation was continued with rescaled growth rates.

### Significance analysis

Significance analysis was performed using the statistical programming language R and R Studio and the included ‘stats’ library. First, all distributions were tested for normality with the ‘shapiro.test’ function. Then, significance analysis was performed depending on the result of the Shapiro test. A two sample t-test was performed for normal distributions using the ‘t.test’ function. A Mann–Whitney–Wilcoxon test, using the ‘wilcoxon.test’ function was performed for distributions with non-normality. The p-value and the U-statistic with the sample sizes of both samples are given in the SI data file.

## Supplementary information

Supplementary information.

Supplementary dataset

## Data Availability

All data generated or analyzed during this study are included in this article (and its Supplementary Information files).
