# Supplementary material for: Dynamics of ColicinE2 production and release determine the competitive success of a toxin-producing bacterial population
Source: Sci Rep. 2020 Mar 4;10:4052. doi: 10.1038/s41598-020-61086-z (PMC7055308; doi:10.1038/s41598-020-61086-z)
Supplement: Supplementary file 1 — Supplementary information. [file 41598_2020_61086_MOESM1_ESM.pdf]

Supplementary Material for

Title: Dynamics of ColicinE2 production and release determine the competitive success of a toxin producing bacterial population

Authors: Anna S. Weiß, Alexandra Götz, Madeleine Opitz

Supplementary Figures

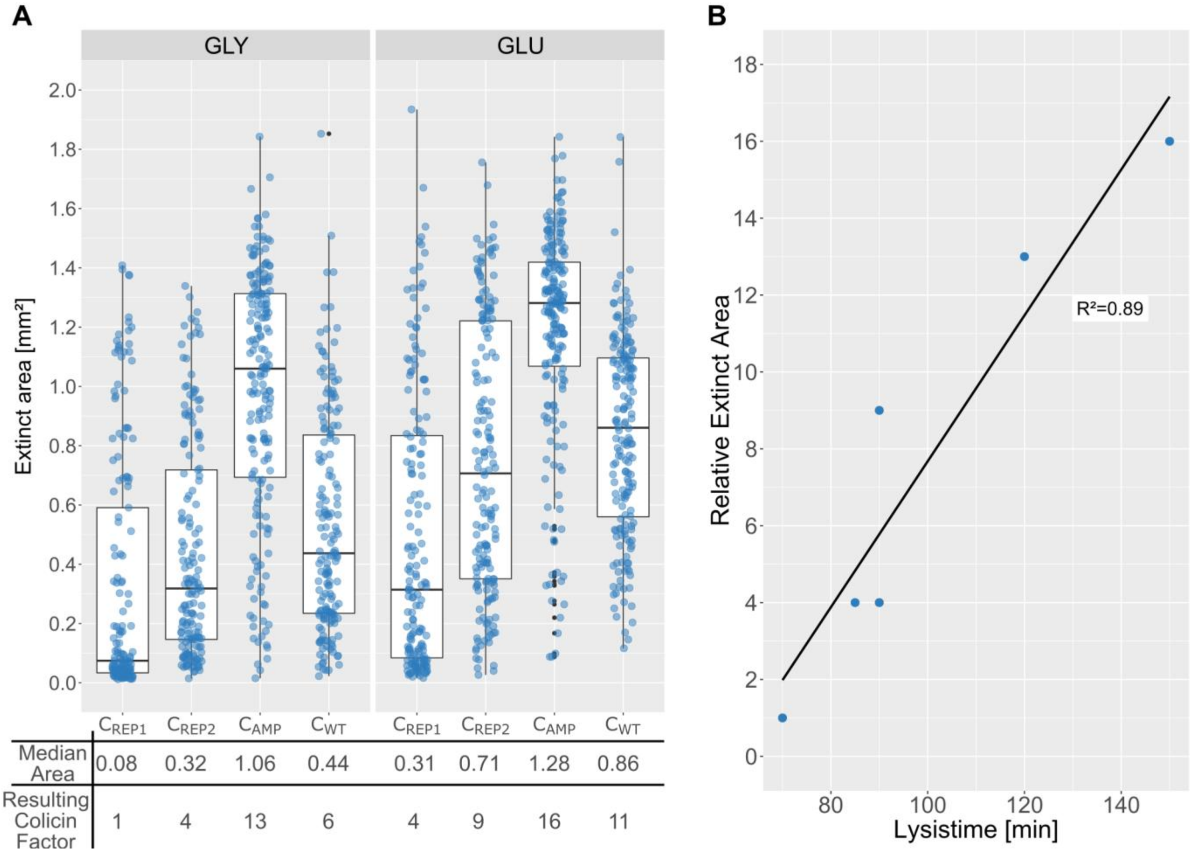

Supplementary Figure 1: Amount of toxin released by different toxin producing strains. A) Toxin release by toxin-producing strains leads to cell death of the S strain. The amount of toxin being released can be quantified by the area of the plated S strain colony that goes extinct after a certain time. The greater the extinct area, the more toxin has been released by the particular C_X strain. The figure shows the extinct S strain area for toxin release by different toxin-producing strains. Details on the experimental procedure can be found in the

Methods section of the main manuscript. The table below the plot summarizes the mean extinct S area and the resulting colicin factor (ratio of amount of S area extinct by C_{REP1} to amount of S area extinct by C_X, with the amount of S area extinct by C_{REP1} set to 1) used in the numerical simulations. B) Toxin amount released by a C_X strain given as the relative extinct S strain area versus the respective lysis time.

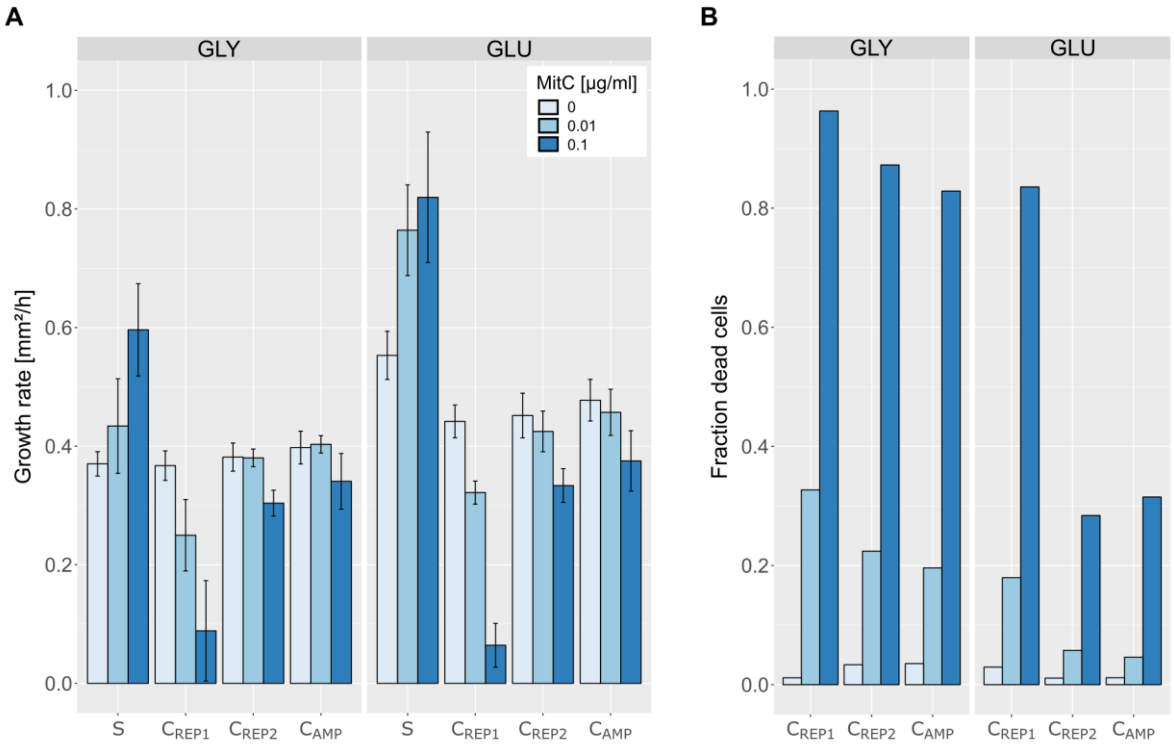

Supplementary Figure 2: Growth rate and number of dead cells of strains used in this study for different concentrations of the SOS inducing agent Mitomycin C grown on either glycerol or glucose as a carbon source. A) Growth rate. Error bars depict the standard deviation. Exact values can be found in the **SI Data** file of this article. B) Fraction of dead cells for C_X strains after 3 hrs of induction with MitC. As the toxin is released upon cell lysis only, the fraction of dead cells directly correlates with the fraction of toxin-producers and is

therefore used to choose the correct parameters in numerical simulations (**Supplementary Fig. 3**).

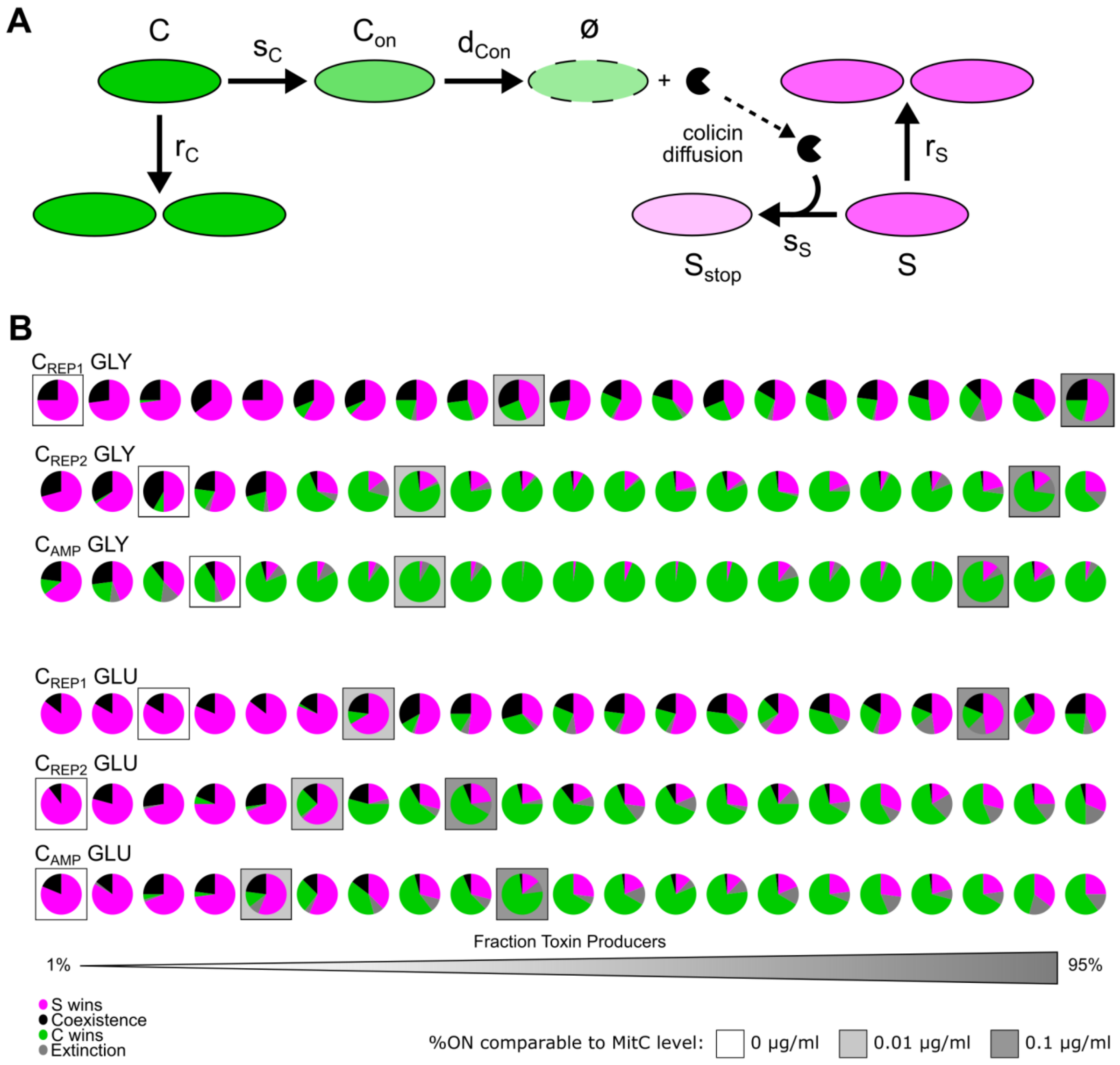

Supplementary Figure 3: Computational model and numerical simulations of the C_X:S competition. A) The interactions scheme underlying the theoretical model **(Methods).** C_X and S strains can reproduce with rate r_C and r_S, respectively. C_X strains can switch into the

toxin-producing state C_{on} with rate s_C. Toxin is released with rate d_{Con} by cell lysis. S cells are inactivated by the toxin with rate s_S, transferring them to the state S_{stop}. B) Numerical simulations of the C_X:S competition given as pie plots of the competition corresponding to 48 hrs. Each row shows the competition of a particular C_X strain. The three upper rows represent simulated competitions with parameters corresponding to experiments performed on glycerol, the three lower rows represent simulated competitions with parameters corresponding to experiments performed on glucose. Simulations are shown for different switching rates, thus increasing the fraction of toxin producers from left to right from 1% to 95%. The boxes around the pie plots indicate those simulations that have a similar fraction of toxin producers as obtained by live-dead experiments (see **Supplementary Fig. 2, Methods**). These pie plots are then compared to the experimental competition data shown in **Fig. 3** in the main manuscript.

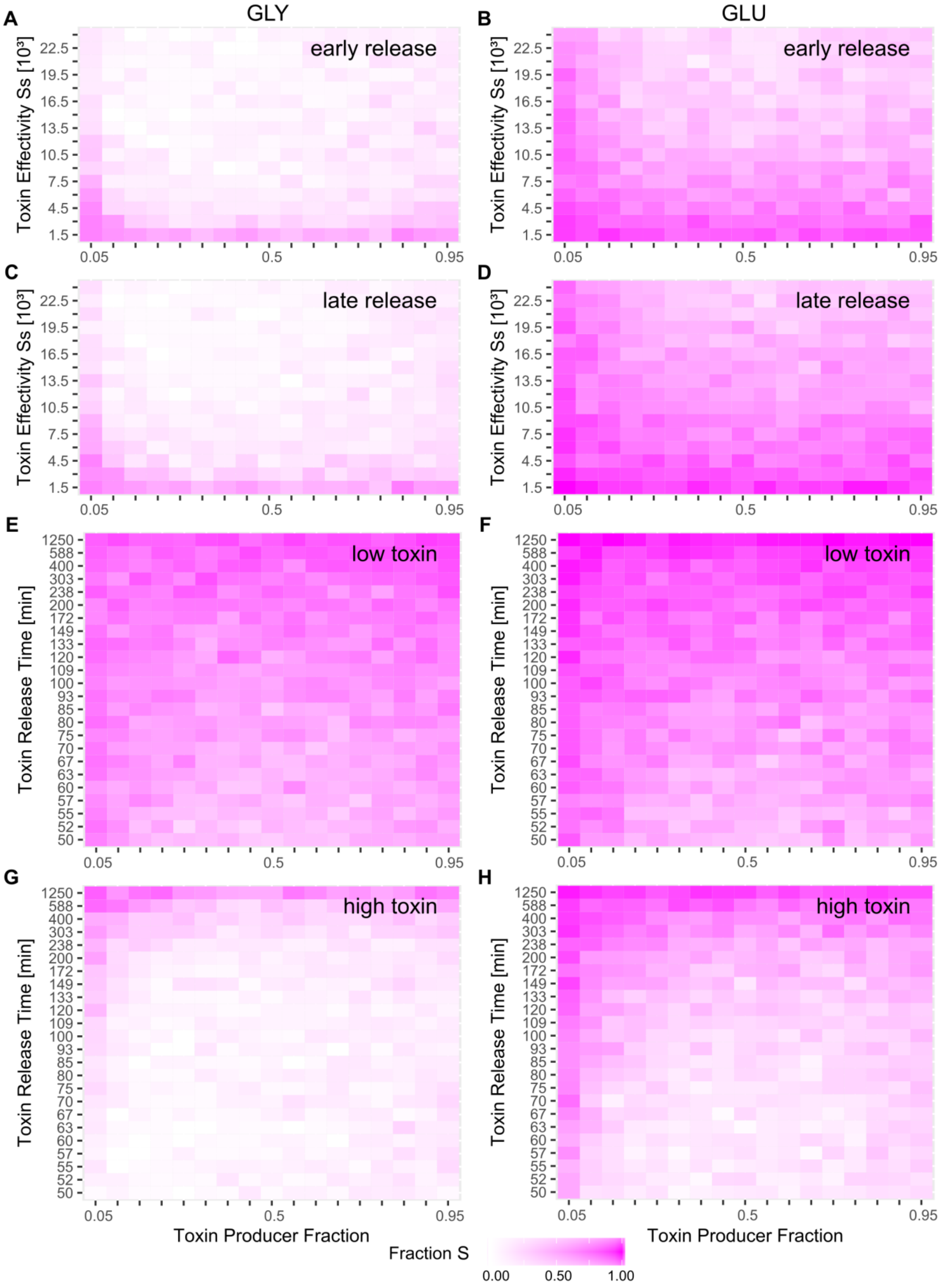

Supplementary Figure 4: Phase diagrams of the fraction S winning the competition.

For different conditions parameter-sweeps were performed in simulations and the fraction

of S strain after 48hrs is plotted. Left column: growth rate of C_{REP1} on glycerol, right column: growth rate of C_{REP1} on glucose-supplemented medium was used for simulation. Toxin release times = 68 min (A) , 90 min (B), 120 min (C), 149 min (D). A-D) Sweep for different toxin effectivities s_S. E) s_S = 1500, F) s_S = 6000, G) s_S = 19500, H) s_S = 24000. E-H) Sweep for varying toxin release times.

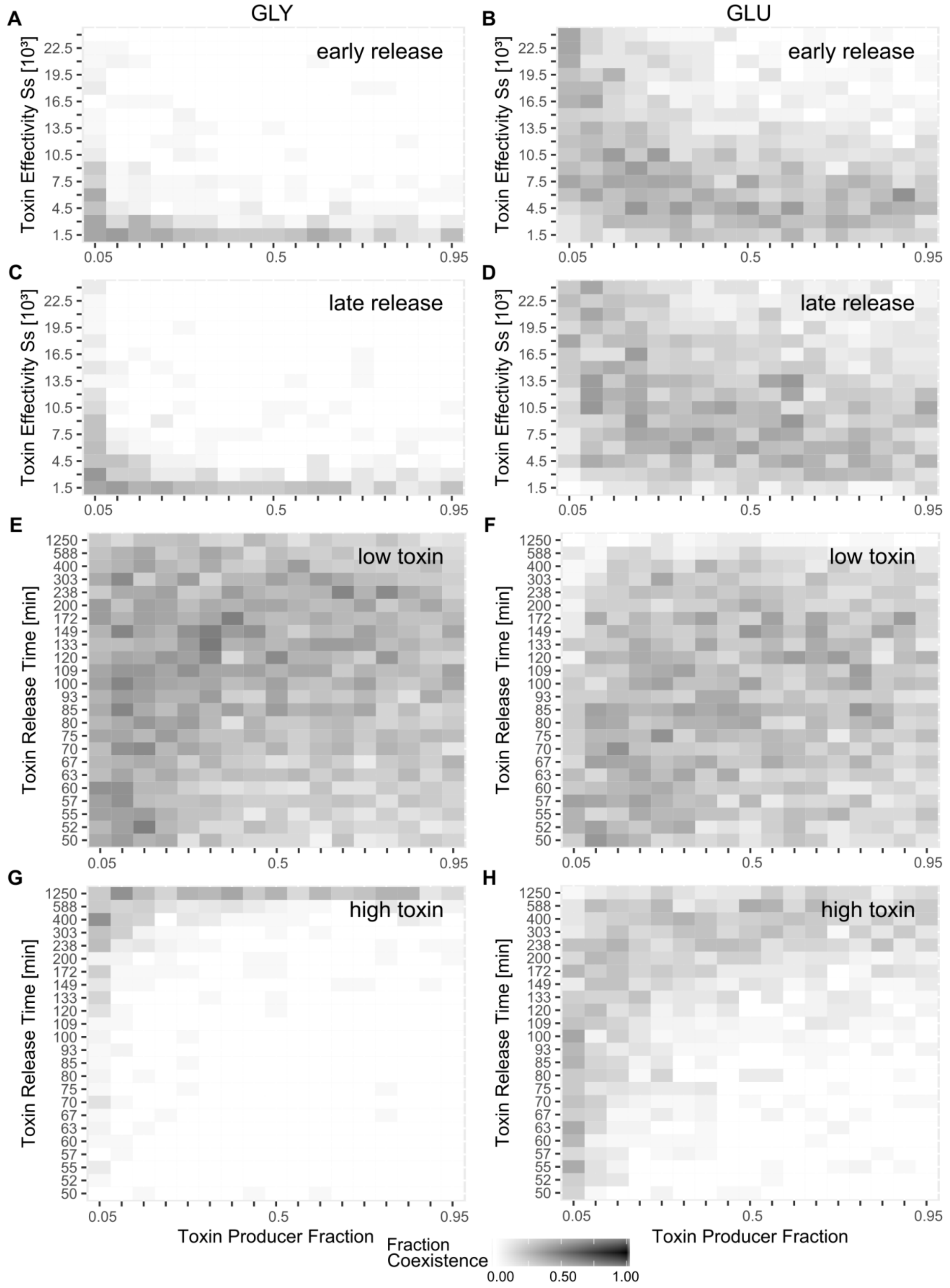

Supplementary Figure 5: Phase diagrams showing the fraction of coexistence. For different conditions, parameter-sweeps were performed in simulations and the fraction of coexistence after 48hrs is plotted. Left column: growth rate of C_{REP1} on glycerol, right

column: growth rate of C_{REP1} on glucose-supplemented medium was used for simulation. Toxin release times = 68 min (A) , 90 min (B), 120 min (C), 149 min (D). A-D) Sweep for different toxin effectivities s_S. E) s_S = 1500, F) s_S = 6000, G) s_S = 19500, H) s_S = 24000. E-H) Sweep for varying toxin release times.

Supplementary Tables

Supplementary Table 1: Bacterial strains used in this study. The table summarizes the genetic details of each strain and lists the plasmids present in each strain. All C strains carry the original ColicinE2 producing pColE2-P9 plasmid. Additionally, C_{REP1} and C_{REP2} carry identical fluorescent reporter plasmids that only differ in their origin of replication resulting in different plasmid copy numbers[1]. Consequently, due to the differences in plasmid composition these two strains differ in the duration of the *cea-cel* delay[1]. The strain C_{AMP} has no additional reporter plasmid, but carries a plasmid providing ampicillin resistance that has no CsrA binding site. Hence, this strain is with respect to its genetic alteration very close to the actual wild-type strain that carries only the pColE2-P9 plasmid and has a lysis-time similar to the wild-type strain.

Strain	Strain description	Information	Reference
C_{WT}	BZB 1011 E2C	Colicin producing strain, carrying pColE2-P9	[2]
C_{REP1}	BZB 1011 E2C + pMO3	Colicin producing strain, carrying pColE2-P9 and the fluorescence reporter plasmid pMO3 with plasmid copy number of ~ 55	[3]
C_{REP2}	BZB 1011 E2C + pMO8	Colicin producing strain, carrying pColE2-P9 and the fluorescence reporter plasmid pMO8 with plasmid copy number of ~ 13	[1]
C_{AMP}	BZB 1011 E2C + pMO9	Colicin producing strain, carrying pColE2-P9 and a plasmid expressing only ß-lactamase to enable resistance against ampicillin. This additional plasmid carries no CsrA binding site.	This study
S_{RFP}	BZB 1011 + pBAD24-mCherry	Colicin sensitive strain with arabinose inducible mCherry-fluorescence reporter plasmid	[4]

Supplementary Table 2: Primers used in this study for the creation of C_{AMP}.

Name	Sequence	Purpose
P1	5´- ATTATGGTACCCAAGCTTGGCTGTTTTGG - 3´	Amp Plasmid [fwd] cloning
P2	5´- ATGTGGTACCGATAAGCTGTCAAACATGAGCAGAT - 3´	Amp Plasmid [rev] cloning

Supplementary Table 3: Parameters used for theoretical modelling and simulations.

Further information can be found in the **SI Data table**.

		SRFP	CREP1	CREP2	CAMP
**GLY**	**GR**	0.068 ±0.004	0.068 ± 0.005	0.070 ± 0.005	0.073 ± 0.006
	**dCon**		0.015	0.012	0.008
**GLU**	**GR**	0.102 ± 0.008	0.081 ± 0.006	0.083 ± 0.007	0.088 ± 0.007
	**dCon**		0.011	0.011	0.007

Supplementary Table 4: Significance analysis corresponding to figure 2. ***:p<0.01, **:p<0.05, n.s.: not significant. A Mann-Whitney-Wilcoxon test was performed to test for significant difference between the distributions.

Ton cea	Crep2-GLY	Crep1-GLU	Crep2-GLU
Crep1-GLY	n.s.	***	
Crep2-GLY			n.s.
Crep1-GLU			**

T lysis	Crep2-GLY	Camp-GLY	Crep1-GLU	Crep2-GLU	Camp-GLU
Crep1-GLY	***	***	***		
Crep2-GLY		***		**	
Camp-GLY					***
Crep1-GLU				n.s.	***
Crep2-GLU					***

Ton cel	Crep2-GLY	Crep1-GLU	Crep2-GLU
Crep1-GLY	***	***	
Crep2-GLY			**
Crep1-GLU			n.s.

Crep1-GLY	Ton cel	T lysis
Ton cea	***	***
Ton cel		***

Crep2-GLY	Ton cel	T lysis
Ton cea	***	***
Ton cel		***

Crep1-GLU	Ton cel	T lysis
Ton cea	***	***
Ton cel		***

Crep2-GLU	Ton cel	T lysis
Ton cea	***	***
Ton cel		***

Supplementary Table 5: Significance analysis corresponding to figure 3. ***: p<0.01, *:p<0.1, n.s.: not significant. A Mann-Whitney-Wilcoxon test was performed to test for significant difference between the distributions.

		GLY-GLY	GLU-GLU
CREP1-0MitC	CREP2-0MitC	***	***
CREP1-0MitC	CAMP-0MitC	n.s.	n.s.
CREP1-0MitC	CREP1-0.01MitC	***	***
CREP1-0MitC	CREP1-0.1MitC	n.s.	***
CREP2-0MitC	CAMP-0.1MitC	***	***
CREP2-0MitC	CREP2-0.01MitC	***	***
CREP2-0MitC	CREP2-0.1MitC	***	***
CAMP-0MitC	CAMP-0.01MitC	***	***
CAMP-0MitC	CAMP-0.1MitC	***	***
CREP1-0.01MitC	CREP2-0.01MitC	*	***
CREP1-0.01MitC	CAMP-0.01MitC	***	***
CREP1-0.01MitC	CREP1-0.1MitC	***	n.s.
CREP2-0.01MitC	CAMP-0.01MitC	***	***
CREP2-0.01MitC	CREP2-0.1MitC	***	***
CAMP-0.01MitC	CAMP-0.1MitC	***	***
CREP1-0.1MitC	CREP2-0.1MitC	***	***
CREP1-0.1MitC	CAMP-0.1MitC	***	***
CREP2-0.1MitC	CAMP-0.1MitC	***	n.s.

GLY	GLU	
CREP1-0MitC	CREP1-0MitC	***
CREP2-0MitC	CREP2-0MitC	***
CAMP-0MitC	CAMP-0MitC	***
CREP1-0.01MitC	CREP1-0.01MitC	n.s.
CREP2-0.01MitC	CREP2-0.01MitC	***
CAMP-0.01MitC	CAMP-0.01MitC	n.s.
CREP1-0.1MitC	CREP1-0.1MitC	***
CREP2-0.1MitC	CREP2-0.1MitC	n.s.
CAMP-0.1MitC	CAMP-0.1MitC	***

Supplementary Table 6: Comparison of differences in the outcome of experimental and simulation results shown in figure. 3 C) and D). Present outcomes in experiment and simulation are marked as grey tiles. Numbers in tiles depict the fraction of the respective outcome in either experiment or simulation (black, bold: main outcome, light grey: other outcomes).

GLY

	0µg/ml MitC						0.01µg/ml MitC						0.1µg/ml MitC					
	CREP1		CREP2		CAMP		CREP1		CREP2		CAMP		CREP1		CREP2		CAMP	
	Exp	Sim	Exp	Sim	Exp	Sim	Exp	Sim	Exp	Sim	Exp	Sim	Exp	Sim	Exp	Sim	Exp	Sim
Coexist.	29%	25%	2%	42%	27%	8%	25%	31%	22%	2%	33%		6%	25%	2%	2%		
C wins				1%		42%	**27%**	25%	**78%**	**79%**	**52%**	**92%**	35%	21%	**93%**	**71%**	**96%**	**81%**
Extinct.				8%		6%				2%		6%	4%	2%		13%	2%	6%
S wins	**71%**	**75%**	**98%**	**50%**	**73%**	**44%**	14%	**44%**		17%	16%	2%	**55%**	**52%**	5%	15%	2%	13%

GLU

	0µg/ml MitC						0.01µg/ml MitC						0.1µg/ml MitC					
	CREP1		CREP2		CAMP		CREP1		CREP2		CAMP		CREP1		CREP2		CAMP	
	Exp	Sim	Exp	Sim	Exp	Sim	Exp	Sim	Exp	Sim	Exp	Sim	Exp	Sim	Exp	Sim	Exp	Sim
Coexist.	16%	17%	1%	10%	15%	19%	29%	23%	**71%**	13%	**58%**	23%	9%	19%	3%	6%	2%	2%
C wins							**67%**	10%	21%	23%	42%	4%	**45%**	17%	**84%**	**60%**	**84%**	**75%**
Extinct.										2%			9%	17%	2%	10%	2%	8%
S wins	**84%**	**83%**	**99%**	**90%**	**85%**	**81%**	5%	**67%**	8%	**63%**		**73%**	36%	**48%**	11%	23%	13%	15%

References

1 Gotz, A. *et al.* CsrA and its regulators control the time-point of ColicinE2 release in Escherichia coli. *Scientific reports* **8**, 6537, doi:10.1038/s41598-018-24699-z (2018).

2 Kerr, B., Riley, M. A., Feldman, M. W. & Bohannan, B. J. Local dispersal promotes biodiversity in a real-life game of rock-paper-scissors. *Nature* **418**, 171-174, doi:10.1038/nature00823 (2002).

3 Mader, A. *et al.* Amount of colicin release in Escherichia coli is regulated by lysis gene expression of the colicin E2 operon. *PloS one* **10**, e0119124, doi:10.1371/journal.pone.0119124 (2015).

4 Weber, M. F., Poxleitner, G., Hebisch, E., Frey, E. & Opitz, M. Chemical warfare and survival strategies in bacterial range expansions. *Journal of the Royal Society, Interface* **11**, 20140172, doi:10.1098/rsif.2014.0172 (2014).
